# Putting the Brakes on Tumorigenesis with Natural Products of Plant Origin: Insights into the Molecular Mechanisms of Actions and Immune Targets for Bladder Cancer Treatment

**DOI:** 10.3390/cells9051213

**Published:** 2020-05-13

**Authors:** Qiushuang Wu, Janet P. C. Wong, Hang Fai Kwok

**Affiliations:** Cancer Centre, Faculty of Health Sciences, University of Macau, Avenida de Universidade, Taipa, Macau; yb97632@um.edu.mo (Q.W.); jan.wongpc@yahoo.com (J.P.C.W.)

**Keywords:** bladder cancer, natural products, plants, apoptosis, immunomolecular targets

## Abstract

Bladder cancer is the 10th most commonly diagnosed cancer worldwide. Although the incidence in men is 4 times higher than that in women, the diagnoses are worse for women. Over the past 30 years, the treatment for bladder cancer has not achieved a significant positive effect, and the outlook for mortality rates due to muscle-invasive bladder cancer and metastatic disease is not optimistic. Phytochemicals found in plants and their derivatives present promising possibilities for cancer therapy with improved treatment effects and reduced toxicity. In this study, we summarize the promising natural products of plant origin with anti-bladder cancer potential, and their anticancer mechanisms—especially apoptotic induction—are discussed. With the developments in immunotherapy, small-molecule targeted immunotherapy has been promoted as a satisfactory approach, and the discovery of novel small molecules against immune targets for bladder cancer treatment from products of plant origin represents a promising avenue of research. It is our hope that this could pave the way for new ideas in the fields of oncology, immunology, phytochemistry, and cell biology, utilizing natural products of plant origin as promising drugs for bladder cancer treatment.

## 1. Introduction

Cancer is an urgent global challenge and has become one of the leading causes of death worldwide. Although clinical treatment has improved during the past decades, the incidence of cancer has experienced rapid growth over the years. In 2018, GLOBOCAN estimated that there were 18.1 million new cancer cases and 9.6 million cancer deaths worldwide, which represents an approximately 30% and 20% increase in incidence and mortality, respectively, in comparison with data for 2012 [1,2]. This undesired growth rate serves as a warning to actively investigate and develop anticancer research.

Bladder cancer is the 10th most commonly diagnosed cancer worldwide, with more than 500,000 new cases of bladder cancer estimated in 2018 and 200,000 deaths, with the cancer being more common in men than women [1]. It was reported that the incidence of bladder cancer in men is about 3–4 times higher than in women; the reasons for this observed gender difference include the tobacco smoking rate, differences in metabolic detoxification of carcinogens, and sex steroids. Among these factors, tobacco smoking is not only the main risk factor for bladder cancer but is also responsible for 50% of all bladder cancer cases. When diagnosed, women with bladder cancer have more advanced tumors and a higher mortality rate. One of the reasons for this is the differences in the biologic pathways between men and women; another main cause may be that the initial diagnosis of women does not occur as early as that of men. Although the mortality rate has decreased in North America and some European and Baltic countries, in the growing economies of Central and South America and some Central and Eastern European countries, no decrease in mortality has been observed over the last few decades [3,4,5].

The continuous study of anticancer drugs and the development of technologies including high-throughput screening, modern molecular biology methods, structure-based target drug design, nanotechnology, and the sequencing of the human genome have tremendously improved drug discovery and assisted the search for new strategies of anticancer therapy [6,7]. The traditional methods—surgery, radiotherapy, and chemotherapy—began to be used in the early 20th century and have become the most common types of cancer treatment. With the arrival of nanotechnology, the applications of nanostructures as new therapeutic alternatives for controlled drug delivery promote the development of cancer therapy and the provision of targeted therapy. In recent years, immunotherapy—and, in particular, immune checkpoint therapy—has provided a novel therapeutic strategy for the treatment of cancer and is now a beneficial therapeutic option in many cancer cases [8]. The 2018 Nobel Prize in Medicine was awarded to two cancer immunotherapy researchers, James P. Allison and Tasuku Honjo, for their discovery of a cancer therapy involving the inhibition of negative immune regulation, which has initiated a revolution in cancer therapy. In addition, the advancements in human genomics have meant that gene therapy has become another promising therapeutic method. From 1949 to 2014, the FDA approved 150 medicines comprising 61 cytotoxic drugs and 89 targeted drugs that were indicated for at least one type of cancer [7]. Cancer treatment has experienced a significant breakthrough over the past century; however, there is still great potential for us to combat this aggressive disease and attempts to discover effective anticancer drugs should never cease.

When we discuss the resources for drug development, natural products must be mentioned. According to the statistics from 1981 to 2018, over 50% of anticancer drugs stem from or are derived from natural products [9]. In the field of natural products, many compounds from plants, marine life, and microorganisms have been developed as anticancer agents. Among these, plants have played a vital role in drug discovery, representing the major source of natural products for human use. Plant resources in nature are extremely rich, but only 10% of the nearly 75,000 higher plant species have been used in traditional medicine, and only 1% to 5% have been studied scientifically [10]. Although plant-derived anticancer agents such as paclitaxel and vincristine along with their semisynthetic analogs have been effectively used for cancer therapy, including bladder cancer, and many synthetics or semi-synthetics based on potential anticancer natural products have been successfully approved, such as topotecan, irinotecan, and the analog of camptothecin, there is still enormous potential for us to explore new possibilities in developing anticancer drugs that are only cytotoxic toward cancer cells.

**Figure 1:**
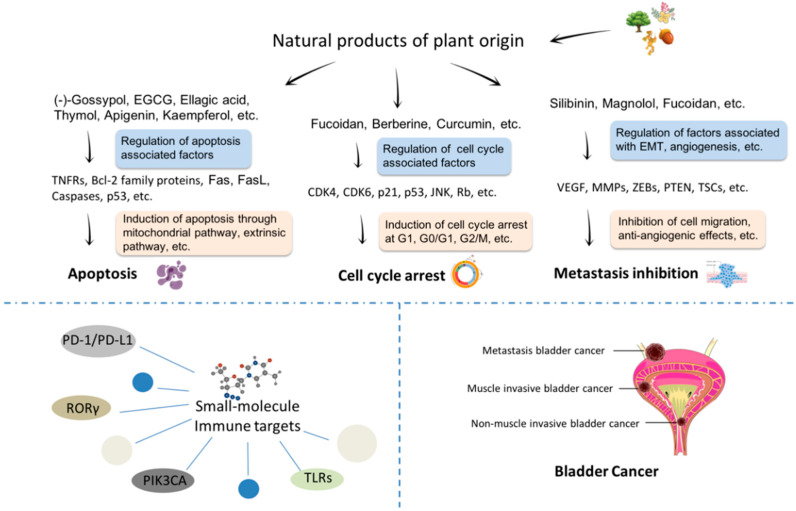
The main mechanisms and potential small-molecule immune-targets of promising plant-origin natural products for bladder cancer treatment.

This review summarizes the promising natural products derived from plants and discusses their anti-bladder cancer mechanisms, including inducing apoptosis, regulating the cell cycle, and anti-metastasis properties (Figure 1). Furthermore, this review includes the discovery of novel small molecules to target immune targets for bladder cancer treatment from products of plant origin in the hope that this might pave the way for new ideas in the fields of oncology, immunology, phytochemistry, and cell biology utilizing natural products of plant origin as promising drugs for bladder cancer treatment.

## 2. Bladder Cancer Treatment

According to the histological diagnosis, bladder cancer can be divided into non-muscle-invasive bladder cancer (NMIBC) and muscle-invasive bladder cancer (MIBC). This classification is very important as it determines the prognosis and treatment. The general treatment includes surgery, intravesical therapy, radical therapy, immunotherapy, and radiotherapy. Different treatments are chosen according to the evaluation of cancer risk, i.e., whether the tumor may return or spread beyond the lining of the bladder [11].

For NMIBCs, the most common treatment is transurethral resection of bladder tumors, in which the tumor is removed and prevented from invading the muscle lining of the bladder, and this kind of cancer is often treated by intravesical therapy in combination with chemotherapy or immunotherapeutic drugs that can help to reduce the rates of recurrence and progression. Mitomycin-C, gemcitabine, and thiotepa are the drugs most often used for intravesical chemotherapy. Other drugs that are used include cisplatin, doxorubicin, and valrubicin. For high-risk tumors, adjuvant Bacillus Calmette–Guérin (BCG) intravesical installations are the standard treatment. Although endoscopic surgical treatment and the administration of BCG help to decrease the tumor progression risk, more than 50% of NMIBC patients are likely to undergo recurrence and experience bladder cancer progression [12].

About 30% of bladder cancers are initially diagnosed as MIBCs, and approximately 10–15% of recrudescent NMIBCs can progress to MIBCs. The essential treatment options include radical cystectomy with or without systemic chemotherapy and chemotherapy with radiation [13]. When compared with locoregional treatment, platinum-based combination neoadjuvant chemotherapy can improve survival outcomes. The most common regimens for systemic or whole-body chemotherapy to treat bladder cancer are as follows: (a) Cisplatin and gemcitabine, (b) carboplatin and gemcitabine, and (c) MVAC, which is a combination of methotrexate, vinblastine, doxorubicin, and cisplatin [14,15,16]. While chemotherapy represents the standard treatment for unresectable and metastatic bladder cancer, there is still a large number of patients who are not compatible with cisplatin-based chemotherapy, and the long-term survival rate remains low. With developments in immunotherapy research, PD-L1, PD-1, and CTLA-4 immune checkpoints have been investigated; five immune checkpoint inhibitors have gained FDA approval as second-line therapeutic regimens, namely atezolizumab, pembrolizumab, avelumab, nivolumab, and durvalumab. Among them, atezolizumab and pembrolizumab were subsequently approved for first-line treatment [17]. One of the highlights of immunotherapy is the investigation of immune checkpoint inhibitors to be used against progressed bladder cancer, and the concurrent targeting of canonical signaling pathways is another focus. An oral pan-fibroblast growth factor receptor (FGFR) agent, erdafitinib, has recently received approval by the FDA for use in the monotherapy of bladder cancer patients whose tumors have a certain FGFR genetic mutation and is locally advanced or metastatic [18].

## 3. Plant-Origin Natural Products in Bladder Cancer Treatment

Patients diagnosed with NMIBC follow the standard treatment, undergoing transurethral resection and intravesical chemotherapy or immunotherapy with BCG. If the tumor invades the muscle or progresses, a combined therapy approach involving neoadjuvant chemotherapy, surgery, immune checkpoint inhibitors, and FGFR inhibitors is recommended. Although the optimal choice helps to decrease the potential for micrometastasis and increases bladder cancer survival outcomes, there are a large number of patients who do not respond to platinum-based chemotherapy and immunotherapy. The development of new treatment methods is necessary for improving outcomes.

The advanced understanding of bladder cancer attained by the comprehensive analysis of genes has provided new options for treatment. FGFR, EGFR, HER2, and VEGF alterations are widely present in bladder cancer patients, and their expression is linked not only to the tumor stage but also to patient survival. Various preclinical studies have reported that EGFR inhibitors showed clear antiproliferative and antiangiogenic effects, and combination therapy using HER2 and VEGF inhibitors has helped to improve outcomes [19]. Numerous FGFR inhibitors are currently undergoing clinical trials for bladder cancer treatment, such as rogaratinib, infigratinib, pemigatinib, and debio 1347, as single or combination agents [20]. The Cancer Genome Atlas (TCGA) has revealed specific mutations and typical signaling pathways associated with bladder cancer, including for the p53/cell cycle, DNA repair, PI3K/AKT, RTK/MAPK, and chromatin modifications and regulation, which were reflected in the analysis of a bladder cancer cohort [21].

Plants are the largest source of small-molecule natural products and should play a vital role in the search for anti-bladder cancer agents; in this review, we summarize the promising plant-origin compounds with their anti-bladder cancer mechanisms (Table 1) in the hope that this could provide insights and innovation for further research studies.

### 3.1. Plant-Origin Compounds Induce Apoptosis in Bladder Cancer

Apoptosis, a type of programmed cell death, is an effective way for cells to die and contribute to the maintenance of the internal environment. In cancer, however, the balance between cell death and cell division is broken, and cells are able to escape apoptosis and continue to divide under abnormal conditions [22].

It must be considered that the escape from apoptosis is a key factor for tumor cell survival, progression, and metastasis. Some research studies have pointed out that many specific apoptotic proteins have been demonstrated in bladder cancer and are associated with tumor stage, invasion, and metastasis [23]. The death receptors TNF receptor (TNFR) and Fas are receptors found on the cell membrane, as well as Fas ligand FasL, which is associated with regulating the apoptosis of extrinsic pathways that receive death signals and activate caspases. Intrinsic mitochondrial pathways are controlled by a group of Bcl-2 family proteins, which can both promote apoptosis and allow apoptosis to continue, leading to DNA damage and p53 activation. Bcl-2 family proteins, including Bax, Bak, Bad, Bcl-Xs, Bid, Bik, Bim, and Hrk, are associated with the promotion of apoptosis, while Bcl-2, Bcl-XL, Bcl-W, Bfl-1, and Mcl-1 inhibit apoptosis. Other apoptotic factors that are released from the mitochondria include the apoptosis-inducing AIF, Smac, DIABLO, and HtrA2. Both intrinsic and extrinsic pathways bring about caspase activation. Caspases form a family of cysteine proteases that are the central regulators of apoptosis and are both the initiators and the executioners. Once the apoptotic program begins, caspases are activated by a subsequent cascade of reactions of apoptotic proteases leading to irreversible apoptosis [24,25,26]. In bladder cancer, Bcl-2 family proteins are more frequently observed in low-grade and low-stage tumors, whereas the expression of both Bcl-XL and Bcl-XS is associated with high-grade and advanced-stage bladder cancer. Moreover, the activity of the proapoptotic protein Bax is not correlated with the apoptosis rate in bladder cancer. It has also been indicated that active caspase-3 and p53 were more frequently found in high-grade and high-stage tumors [27]. The current anticancer strategies, such as chemotherapy, primarily trigger apoptosis in tumor cells to achieve their therapeutic effect. Apoptosis-associated markers may act as small-molecule targets for bladder cancer and play an important role in promoting novel therapeutic agent discovery.

Polyphenols exist widely in vegetables, fruits, soy, nuts, and tea, and present strong antioxidant properties. Many researchers have demonstrated that these compounds exhibit potential anticancer activity by modulating multiple diverse biochemical targets and signaling pathways [28,29,30,31]. Several polyphenol compounds that were reported as potential anti-bladder cancer agents induce tumor cell apoptosis.

Gossypol is a polyphenol derived from cotton plants and has been reported to exhibit anticancer properties in several kinds of tumors, including breast, liver, colon, and prostate cancers [32]. Gossypol is isolated as a racemic mixture and exists in (+) and (−) enantiomers. The antiproliferation effect was reported as being more sensitive and significant for lower concentrations of (−)-gossypol than (+)-gossypol when used to treat cancer cells [33]. As a BH3 mimetic, which specifically targets the Bcl-2 family proteins, (−)-gossypol induces apoptosis in bladder cancer cells through the caspase-mediated signaling pathway and is associated with the activation of caspase-3 and -9, downregulation of Bcl-xl and Mcl-1, and upregulation of Bim and Puma proteins. Moreover, (−)-gossypol also helps to sensitize the bladder cancer cells that are resistant to standard chemotherapeutic drugs and can induce apoptosis [34,35]. As it can be administered orally, the possibility exists that (−)-gossypol could be developed as a bladder cancer chemotherapy agent.

Green tea is one of the most popular drinks worldwide, especially in China, and it has been reported that green tea has health benefits for several ailments, including in being antihypertensive, controlling body weight, lowering cholesterol, and the prevention of cancer. Polyphenols and their representative components may contribute to these effects, with the major component being (−)-epigallocatechin-3-gallate (EGCG) [36]. It was suggested that EGCG might function as a chemoprevention agent in bladder cancer treatment and might be associated with the ability to induce apoptosis in T24 and 5637 cells. When used to treat normal bladder cells, no obvious toxicity was observed. The apoptosis-inducing mechanism of EGCG is associated with the PI3K/Akt signaling pathway. After treatment with EGCG, the Bcl-2 and Bcl-xL proteins were downregulated, while Bax and Bad were upregulated; additionally, EGCG promotes the activation of caspase-3 and led to PARP cleavage [37,38].

Ellagic acid is a polyphenolic compound that is widely found in fruits such as strawberries, blackberries, grapes, and pomegranates, as well as in vegetables and some edible mushrooms in high quantities. Ellagic acid shows anticancer activity in different cancers, including being able to induce cell cycle arrest and inhibit cell migration and angiogenesis; moreover, the anticancer effects are mostly attributed to the suppression of cell proliferation and induction of apoptosis [39]. In bladder cancer, ellagic acid reduced the viability of TSGH8301 bladder cancer cells by the induction of apoptosis, which involved the induction of Bax expression, and then caused mitochondrial dysfunction, cytochrome c release, and activation of caspase-3 and -9, indicating that ellagic acid-induced apoptosis occurs through the mitochondrial pathway [40]. This demonstrated apoptotic induction effect is also observed in T24 cells [41].

Thymol is a monoterpene phenol that is present in the oil fraction of several plants. Thymol and its derivatives are reported to be potential anticancer agents with their antiproliferative, antioxidant, and apoptotic induction effects; at the same time, thymol does not show obvious toxicity in normal cells. When thymol was treated with T24 cells, apoptosis was triggered via the intrinsic mitochondria pathway. The expression levels of Bcl-2, Bcl-xl, and Mcl-1 were reduced in T24 cells in a dose-dependent manner, while Bax expression was up-regulated. The release of cytochrome c and Smac/DIABLO from mitochondria into the cytosol has also been observed [42,43].

Flavonoids are an important class of polyphenol components that have numerous bioactivities, including antioxidant, antibacterial, antivirus, anti-vascular disease, and anticancer effects. Some studies have found that the anticancer effect of flavonoids is correlated with inducing apoptosis, suppressing cell proliferation, and inhibiting angiogenesis. Within anti-bladder cancer studies, some flavonoids have shown an excellent effect as potential agents by inducing cell apoptosis [44].

Apigenin, the most common flavonoid, is widely found in fruits and vegetables; parsley, celery, celeriac, and chamomile tea are the most common sources. It has been demonstrated that apigenin could not only help to improve cardiovascular conditions but also stimulates the immune system and shows great potential for the prevention and treatment of many different kinds of cancers [45]. Apigenin can induce dose- and time-dependent cell death and apoptosis and inhibit the migration and invasion of T24 bladder cancer cells. Apigenin leads to apoptosis through a mitochondrial pathway that involves the PI3K/Akt pathway. After treatment with apigenin, the expression of Bax, Bad, and Bak increased, while Bcl-2, Bcl-XL, and Mcl-1 decreased. It also indicates that apigenin promoted the activation of caspase-3, -7, and -9 and led to the cleavage of PARP, which is the major indicator of apoptosis [46,47]. Changes in the proapoptotic and antiapoptotic proteins of the Bcl-2 family and the activation of caspase-3, 7, 9, and PARP suggested that apigenin is a potential chemotherapeutic agent.

Another flavonoid—kaempferol—is found in a variety of plants such as *Hamamelis mollis* Oliver, tea, grapefruit, ginger, and broccoli. The anticancer ability of kaempferol is related to apoptosis, cell cycle arrest, anti-angiogenesis, and anti-metastasis [48,49]. Kaempferol-induced apoptosis in bladder cancer is associated with the PI3K/Akt signaling pathway; the expression of anti-apoptotic proteins was downregulated, while pro-apoptotic proteins were upregulated. Meanwhile, the total levels of p53 slightly decreased [50]. Kaempferol also shows minimal side effects when combined with other chemotherapeutic drugs, which would help to promote this new combination therapy in bladder cancer [51].

Baicalein, a kind of phenolic flavonoid, is isolated from the roots of *Scutellaria baicalensis* and has also shown the ability to induce apoptosis in bladder cancer cells. When T24 cells were treated with 100 μM baicalein, the upregulation of p16, p21, and Bax and cleavage of both caspase-3 and -9 were observed, along with the downregulation of Bcl-2. In normal bladder cells, no significant effects were observed for the same concentration of baicalein [52].

Curcumin is one of the main ingredients in *Curcuma* spp. plants and is commonly used as a coloring agent and safe food additive. Researchers have been investigating the strong anticancer ability of curcumin demonstrated in several cancer cell lines, including bladder cancer [53,54]. Curcumin suppressed cell proliferation of several bladder cancer cells by inducing apoptosis, but the mechanism still needs to be elucidated [55,56,57,58].

Kazinol A is derived from *Broussonetia papyrifera*, which is used as traditional Chinese medicine. Kazinol A showed higher cytotoxicity than other *Broussonetia papyrifera* origin flavonols in bladder cancer cells, including drug-resistant cells. Kazinol A decreased the phospho-AKT levels, which could induce a decrease of phospho-Bad, resulting in the inhibition of anti-apoptotic proteins Bcl-2 and BCL-XL. This compound crosses the mitochondrial membrane and inhibits phospho-Bad, resulting in the induction of apoptosis in T24 and T24R2 cells in a mechanism that may be associated with the AKT signaling pathway [59].

Alkaloids play a vital role in the history of anticancer drug development; camptothecin, paclitaxel, vinblastine, and vincristine and their semi-synthetic analogs have been used in clinical treatment for over 30 years. While able to kill tumors by inhibiting DNA topoisomerases, which leads to DNA damage, and inhibiting tubulin polymerization, which leads to the prevention of mitotic spindle formation, other observed side effects constituted the main barrier for their further use [60]. Alkaloids are continuously discovered, and several of these have been shown to be potent modulators of apoptosis in bladder cancer cells; these investigations present new insights for bladder cancer treatment.

Boldine is one of the alkaloids isolated from different parts of *Peumus boldus*, especially the leaves and bark. This outstanding alkaloid not only has hepatoprotective, cytoprotective, anti-inflammatory, and choleretic properties but also been found to present an antiproliferative ability in cell lines of breast cancer, liver cancer, and bladder cancer [61]. In T24 bladder cancer cells, boldine-induced apoptosis is correlated with activation of the ERK and ATK signaling pathways [62].

Lycorine, extracted from the *Amaryllidaceae genera*, was demonstrated to have anti-bladder cancer activity by inducing apoptosis; the effect was mediated by inhibiting phospho-Akt expression and activating caspase-3 and Bax, as demonstrated in vitro. Consequently, lycorine also inhibited tumor growth in vivo [63].

Tetrandrine exists in the rhizomes of *Stephania tetrandra*. This bisbenzylisoquinoline alkaloid has been used in clinical trials to treat arthritis, rheumatism, hypertension, and inflammation. Some researchers have shown that tetrandrine also possesses an anticancer effect. It was observed that tetrandrine inhibited the T24 and 5637 bladder cancer cells. A total of 48 h of tetrandrine treatment at 20 μM resulted in 71.7% of apoptotic cells in 5637 cell lines, and a similar effect was observed in T24 cell lines. Caspase-8 and -9 were activated, while caspase-3 was induced by tetrandrine treatment; furthermore, the release of cytochrome c was observed, accompanied by the collapse of Δψ_m_, suggesting that tetrandrine induced-apoptosis was associated with the mitochondrial pathway [64].

Other kinds of natural compounds of plant origin have also been revealed to have an ability to induce apoptosis and thus are considered potential anticancer agents for bladder cancer.

6’-Hydroxy justicidin A, isolated from plant *Justicia procumbens*, has a similar molecular structure to podophyllotoxin derived from clinical anticancer agents such as etoposide and teniposide, and is cytotoxic in bladder cancer EJ cells. This lignan compound increased the enzymatic activity of caspase-3, -8, and -9, which is associated with the initiation of the mitochondrial apoptotic pathway [65].

Fucoidan is a polysaccharide isolated from algae and seaweeds. These sea plants have been used in healthcare medicine and as supplements in functional foods. After treatment with fucoidan, Fas, the key extrinsic pathway, was upregulated and there was a loss of MMP; then, cytochrome c was released from mitochondria, promoting the activation of caspases, and concurrently mediating Bcl-2 and IAP family members, leading to the apoptosis of T24 cells, which was related to both intrinsic and extrinsic apoptotic signaling pathways [66]. In the human bladder cancer cell line 5673, fucoidan-induced apoptosis occurred via the PI3K/Akt signaling pathway [67,68].

### 3.2. Plant-Origin Compounds Induce Cell Cycle Arrest in Bladder Cancer

The cell cycle is a cyclic process that leads to DNA replication and the division of cytoplasm and organelles to produce two daughter cells. [69]. Cyclins and cyclin-dependent kinases (CDKs) are central to the mechanism by which cells progress through the cell cycle. The activation of CDKs is positively regulated by binding to the cyclins and negatively regulated by the activity of CDK inhibitors, including the INK4 family proteins p16, p15, p18, and 19, and the Cip/Kip family proteins p21, p27, and p57 [70]. Aberrations in cell cycle regulation are one of the most extensively investigated molecular aspects of bladder cancer. p53 and Rb pathways, as well as CDKs, which primarily control the cell cycle, are frequently altered in bladder cancer. Patients with mutated p53 showed significantly decreased survival times compared with those with wild type p53. On the contrary, p21, downstream of p53, shows decreased expression in advanced bladder cancer. Patients with negative p21 and altered p53 tumors have a higher risk of recurrence and lower survival rate when compared to those with positive p21. Since the phosphorylation of members of the retinoblastoma (Rb) protein family is associated with CDK4,6 activation, the inhibition of CDK4,6 results in Rb dephosphorylation, which leads to G0/G1 arrest. About 80% of bladder cancer patients maintain functional Rb1, which is considered as a promising target [27,71]. Anticancer studies focus on the restoration of cell cycle control by modulating molecular targets involved in cancer cell alterations, such as the inhibition of CDKs, downregulation of cyclins, overexpression of endogenous CDK inhibitors, disruption of cyclin/CDK interactions, altered proteolysis, degradation of cyclins, etc. [72].

Fucoidan has been mentioned as inducing bladder cancer apoptosis via the PI3K/Akt signaling pathway; the suppressed cell proliferation effect of fucoidan is also associated with the induction of cell cycle arrest in the G1 phase. Park et al. found that the restriction of E2F expression and the increase in p21 plays a crucial role in bladder cancer G1 cell cycle arrest. Moreover, the increased p21 expression caused by fucoidan was involved in the inhibition of pRB phosphorylation [66,73].

Berberine is famous for its antibacterial effect and is non-toxic to normal human cells. Although berberine is used in clinical practice as an antibacterial agent, its anticancer effect has been indicated for several kinds of cancer cell lines, including breast, lung, colon, and bladder. Bladder cancer cell proliferation was inhibited by berberine treatment in a dose-dependent manner, which was correlated with G0/G1 cell cycle arrest [74]. 

As a widely-studied compound, curcumin also influences the cell cycle progression in bladder cancer cell lines. In T24 cells, p27 was significantly upregulated, while cyclin E1 was suppressed by curcumin, which resulted in the G2/M cell cycle arrest [55]. In 253JB-V and KU7 bladder cancer cell lines, the curcumin-induced cell cycle arrest was in different phases. 5 μmol/L curcumin could inhibit G0-G1 to S phase progression in 253JB-V. In constrast, the same concentration of curcumin can not inhibit cell cycle progression of KU7 cells until increasing to 25 μmol/L [57].

### 3.3. Plant-Origin Compounds with Anti-Bladder Cancer Metastasis Activity

In terms of metastasis—the migration of malignant tumor cells from the site of primary tumors to distant secondary tissues—patients with metastatic bladder cancer have a poor prognosis, and this is a major cause of bladder cancer mortality. Cancer metastasis is a complex regulatory process that involves mobilization, invasion, intravasation, and transit within the vasculature and arrest, extravasation, and colonization [75]. There are three primary ways that tumors can spread to distant organs: The circulatory system, the lymphatic system, and passage through the body wall into the abdominal and chest cavities.

Although the 5-year survival rate of NMIBC is greater than 88%, and that for MIBC which has not yet spread outside the bladder is 70%, this rate decreases to 36% if the tumor extends through the bladder. Furthermore, once the bladder cancer progresses to being metastatic, the 5-year survival rate drops to 5%, and the median survival for patients with metastatic tumor is only 15 months [76,77,78]. The initiation of metastasis requires invasion, which is enabled by the epithelial–mesenchymal transition (EMT). Through EMT, epithelial cells lose their cell polarity and disconnect from the basement membrane, and the cancer cells could then have a higher ability to migrate and invade, resist apoptosis, and degrade the extracellular matrix. An analysis of the major EMT players, including CDHs, CTNNB1, GSK3B, MMP2, MUC1, SNAI1/2, TWIST1, VIM, and ZEB1/2, found that these genes are deregulated in 66% of patients; the results indicated a significant association between overall survival and EMT players in metastatic bladder cancer. In addition, higher expression levels of mesenchymal markers such as MMP2, VIM, TWIST1, and ZEB1/2, were found in high-stage samples [79].

Silibinin is isolated from thistle seeds. In a TGF-β1-induced bladder cancer cell EMT model, the expression levels of mesenchymal markers N-cadherin, vimentin, β-catenin, and ZEB1 were increased, while the expression of E-cadherin decreased; these EMT markers are regulated by COX-2. After treatment with silibinin, the migration and invasion induced by TGF-β1 were suppressed, and the anti-metastatic effect of silibinin was associated with COX-2 downregulation [80]. In another study, silibinin suppressed migration and invasion in a highly metastasized T24-L cell model, targeting the metastatic tumor by inactivating the β-catenin/ZEB1 signaling pathway, which regulated EMT markers and MMP2 to reverse the EMT process in bladder cancer. Moreover, an in vivo study indicated silibinin could decrease the potential for bladder cancer to metastasize to the lung and prolonged animal survival [81].

Tumor growth and metastasis are vascular-dependent, and the ability to generate new blood vessels is recognized as an important marker of the aggressiveness of malignant tumors. Neovascularization provides cancer cells with abundant nutrients, oxygen, and growth factors and is also an important channel for cancer cell metastasis. Therefore, inhibiting tumor neovascularization and cutting off the nutrient supply of the tumor can inhibit tumor growth and reduce the chance of cancer cell invasion and metastasis.

Magnolol, isolated from *Magnolia officinalis*, has been reported to inhibit tumor angiogenesis in bladder cancer both in vitro and in vivo. HIF-1α is one of the most important transcriptional factors that regulate VEGF to promote tumor angiogenesis. Magnolol was considered to be a VEGFR2 antagonist; this compound inhibited HIF-1a and VEGF expression in vitro, leading to inhibition of hypoxia-induced angiogenesis. Meanwhile, it was observed that magnolol suppressed in vivo tumor growth and angiogenesis [82].

Along with inducing apoptosis and cell cycle arrest, an antiangiogenic effect was also found in low molecular weight fucoidan. Low molecular weight fucoidan showed an antiangiogenic ability in human umbilical vascular endothelial cells and inhibited the migration and invasion of T24 cells to distal sites. PI3K/AKT/mTOR are involved in HIF-1α and VEGF expression, and the administration of low molecular weight fucoidan down-regulated the hypoxia-activated phosphorylation of PI3K/AKT/mTOR; hypoxia-induced HIF-1α expression and VEGF secretion were suppressed [83].

**Table 1:** Promising plant origin natural products for bladder cancer therapy.

Plant Origin Compounds	Source	Structure	Mechanisms	Related Factors	Ref.
(−)-Gossypol	Cotton plants	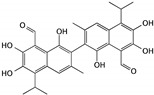	Inducing apoptosis through caspase-mediated signaling pathways	caspase-3, caspase-9, Bcl-xl, Mcl-1, Bim, and Puma	[33,34,35]
(−)-epigallocatechin-3-gallate (EGCG)	Green tea	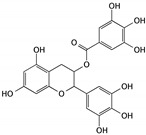	Inducing apoptosis associated with the PI3K/Akt signaling pathway	Bcl-2, Bcl-xL, Bcl-2, Bcl-xL, caspase-3, and PARP	[36,37,38]
Ellagic acid	Fruits and some mushrooms	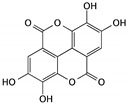	Inducing apoptosis through the mitochondrial pathway	caspase-3, caspase-9, Bax, and cytochrome c	[39,40,41]
Thymol	*Thymus serpyllum* L.	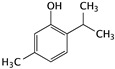	Triggering apoptosis via the intrinsic pathway	Bcl-2, Bcl-xl, Mcl-1, cytochrome c, and Smac/DIABLO	[42,43]
Apigenin	Fruits and vegetables, like parsley, celery, celeriac, and chamomile tea	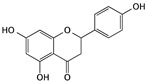	Inducing apoptosis through the PI3K/Akt signaling pathway	Bax, Bad, Bak, Bcl-2, Bcl-XL, Mcl-1, caspase-3, 7, 9, and PARP	[45,46,47]
Kaempferol	*Hamamelis mollis*	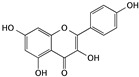	Inducing apoptosis associated with the PI3K/Akt signaling pathway	AKT, Bid, Mcl-1, Bcl-xL, p53, p21, p38, Bax, and Bid	[48,49,50,51]
Baicalein	*Scutellaria baicalensis*	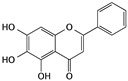	Inducing apoptosis and cell cycle arrest; inhibiting cell migration	p16, p21, p38, CDC2 Bcl-2, Bax, caspase-3, and caspase-9	[52,84]
Curcumin	*Curcuma spp*. plants	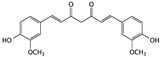	Inducing apoptosis and cell cycle arrest	caspase-3, caspase-7, PARP, Bcl-2, p21, p27, cyclin D1 and cyclin E1	[53,54,55,56]
Kazinol A	*Broussonetia papyrifera*	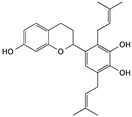	Inducing apoptosis associated with the AKT signaling pathway	p-AKT, p-Bid, Mcl-1, and Bcl-xL	[59]
Boldine	*Peumus boldus*	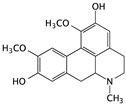	Inducing apoptosis mechanism associated with the ERK and AKT signaling pathway	p-AKT, p-ERK, and p-GSK-3β	[61,62]
Lycorine	*Amaryllidaceae genera*	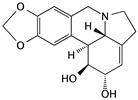	Inducing apoptosis	p-ATK, caspase-3, and Bax	[63]
Tetrandrine	*Stephania tetrandra*	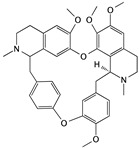	Inducing apoptosis associated with the mitochondrial pathway	caspase-3, caspase-9, and cytochrome c	[64]
6’-hydroxy justicidin A	*Justicia procumbens*	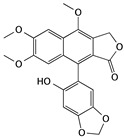	Inducing apoptosis associated with the mitochondrial pathways	caspase-3 and caspase-9	[65]
Fucoidan	Algae and seaweeds	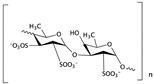	Inducing apoptosis through the PI3K/Akt signaling pathway; inducing cell cycle arrest in the G1 phase; antiangiogenic and inhibiting cell migration and invasion	Fas, MMP, Bax, Bcl-2, cytochrome c, p21, CDK4, CDK6, p-RB, HIF-1α, and VEGF	[66,67,68,73,83]
Berberine	Berberis	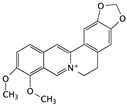	G0/G1 cell cycle arrest and inducing apoptosis	caspase-3 and caspase-9	[74]
Silibinin	Thistle	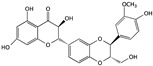	Inhibiting cell migration and invasion	mesenchymal markers, N-cadherin, vimentin, β-catenin, ZEB1, E-cadherin, COX-2, and MMP-2	[80,81]
Magnolol	*Magnolia officinalis*	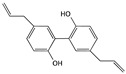	Inhibiting tumor angiogenesis	HIF-1a and VEGF	[82]

## 4. Small-Molecule Immune Targets for Bladder Cancer

Although 75% of bladder cancer patients are initially diagnosed as having NMIBC, more than 50% of them are likely to relapse and progress to a worse diagnosis; disappointingly, MIBC and metastasized bladder cancer are high-grade, with a 5-year overall survival rate of only 15% [21]. Over the past 30 years, the treatment for bladder cancer has not significantly changed until the application of PD-1 and PD-L1 inhibitors in bladder cancer therapy. The application of immunotherapy sheds new light on bladder cancer treatment.

Meanwhile, with the clinical advances made in relation to immune checkpoints, such as in the case of PD-1 and PD-L1, small molecules for immune target therapies have been promoted from preclinical research to undergoing clinical trials. Small-molecule drugs have generally benefited from their specific molecular structures, oral bioavailability, and great invasive ability, able to reach the tumor microenvironment and offer opportunities to traverse cellular membranes and reach intracellular targets that are inaccessible to antibody therapeutics. Some reviews have summarized the small-molecule targets in immuno-oncology, which are currently under investigation, including PD-1/PD-L1, RORγt, chemokine receptor, TGF-β, STING, Toll-like receptors, IDO, arginase, and adenosine receptor. These small molecules target different intracellular pathways and could generally be classified as immune checkpoint blockades, potentiating lymphocyte activation agents and agents that directly target the tumor microenvironment [85,86,87,88].

The immune checkpoint is one of the most investigated immune targets. Despite many monoclonal antibodies having been applied in clinical therapy, small-molecule immune checkpoint inhibitors are appearing in immunotherapy. CA-170 is a PD-L1- and VISTA-targeted small-molecule checkpoint inhibitor, and the only therapeutic agent in clinical trials. In preclinical animal studies, signs of toxicity appeared until oral administration at up to 1000 mg/kg; CA-170 inhibits tumor growth, enhances peripheral T cell activation, and promotes the activation of T cells. It is expected that we will see a clinical response and improvement in the patient survival rate in the near future. In addition, several small-molecule inhibitors of PD-1 and PD-L1 are progressing to the preclinical and/or clinical stages. Sasikumar summarized the small-molecule inhibitors targeted by PD-1/PD-L1 that are currently under preclinical study, and over 30 compounds have been investigated and associated with VISTA, CD-47/SIRPα, and inhibitory receptors on T cells [87,89,90]. Recently, Claudia and colleagues found that ellagic acid showed antiproliferation effects in four bladder cancer cell lines, inhibiting extracellular matrix invasion and migration; moreover, the expression of PD-L1 in UM-UC-3 and T24 cells were downregulated after ellagic acid treatment. These data indicated that ellagic acid, as a potential anti-PD-L1 agent, might contribute to reducing immune escape in bladder cancer [41].

RORγt is considered to be an immune cell master control switch and is mainly expressed in lymphoid tissues; it is critical for the development and maintenance of Th17 and Tc17 T cells, which play a key role in mediating immune responses. RORγt has been considered to be a novel target for cancer immunotherapy. In vitro studies have found that the RORγt agonist could help to improve the survival of Type 17 T cells and enhance the persistence of transferred T cells. Additionally, the conversion of effector T cells into Treg can be limited by RORγt agonists, and T cell exhaustion markers could also be reduced, sustaining the antitumor function of Type 17 T cells. The RORγt agonist improved the effector function of T cells by mediating the expression of IL-17, resulting in the enhancement of lymphocytes. Meanwhile, the RORγt agonist decreased the immune suppression ability by down-regulating the levels of co-inhibitory receptors such as PD-1 [91,92].

Toll-like receptors (TLRs) are membrane receptors that are expressed on the surfaces of both immune and tumor cells, playing a significant role in innate immunity. As innate immunity is associated with the initiation of the immune response against tumor cells, TLRs are considered to be a crucial target in anticancer immunotherapy. Several small molecules have been discovered to be TLR agonists, including imiquimod, resiquimod, S-27609, loxoribine, motolimod, and imidazoquinoline. Among them, imiquimod has acquired FDA approval for clinical use in single-agent therapy for the treatment of cancer [85,87,93].

The PI3K/AKT/mTOR pathway is a well-studied signaling pathway that integrates a number of extracellular targets to regulate cell growth, differentiation, angiogenesis, and cellular metabolism. It is one of the most important signaling pathways and is frequently activated in several cancers, including bladder cancer. Recently, research data have indicated that inhibiting regulators of the PI3K/AKT/mTOR pathway may not only suppress tumor growth but also enhance tumor immunosurveillance by affecting immune cell effectors. It was suggested that PI3Kγ and PI3Kδ play a critical role in the suppression and stimulation of the immune reaction. Inhibiting these two isoforms could lead to decreased degranulation in CD8+ T cells; moreover, inactivating PI3Kγ could restore T cell inhibition [78]. In a PTEN knockout mouse model, the expression levels of immunosuppressive cytokines, chemokines, and angiogenic factors were increased. When lacking the tumor suppressor gene PTEN, T cell-mediated tumor killing was inhibited, and there was decreased infiltration of CD8^+^ T cells into the tumor; at the same time, the overexpression of PD-L1 was observed in both human and mouse tumors [94]. In bladder cancer, it was shown that up to 40% of cases had genetic alterations in components of the PI3K/AKT/mTOR pathway, including PTEN deletions and activating mutations for TSC-1, PIK3CA, and AKT. Either the inactivation of PTEN and TSC1/2 or activation of mutations of PIK3CA are commonly observed in advanced bladder cancer and are associated with worse outcomes [95]. Borcoman and her colleagues were analyzing both NMIBC and MIBC samples and found that a lower expression of the immune signature genes was correlated with the activation of mutations of PIK3CA. They built an animal model to assess the effect of a PI3K inhibitor on T cell infiltration in MIBC and observed an increasing number of immune cells, particularly the CD3^+^ T cells, in tumor infiltration [96]. Inhibitors targeting these critical regulators could be considered as part of the therapeutic strategy to improve the immune response in advanced bladder cancer.

The use of antibodies in immunotherapy has resulted in great progress being made in cancer therapy, with few side effects and high specificity. However, certain limitations have precluded the use of antibodies in immuno-oncology, such as their long half-life, difficulty to penetrate into the tumor, instability during transport and storage, and/or poor large-scale production, which have become obstacles to their wide application in all kinds of patients. Due to the limited immunotherapy strategies available for bladder cancer treatment, small-molecule immune targeting should be considered. Despite being less specific than antibodies, small molecules provide increased possibilities for immunotherapy, and these compounds can reach the tumor microenvironment to activate innate immunity or stimulate immune cells that bio-macromolecules are not able to access. Natural products represent the biggest source of small molecules, providing a great potential for us to develop new agents from compounds of plant origin. Although a few publications exist on the use of natural products as immunotherapy agents, there is still further opportunities to explore plants through the structure–activity simulation technology, molecular modeling-based drug design, and phytochemistry taxology. This might offer more possibilities in the future to discover compounds with broader therapeutic applications in bladder cancer.

## 5. Conclusions

Despite their impressive track record, natural products seem to have fallen out of favor as a source for new drug discovery. The extensive time and effort needed to exploit roots or tree bark to get a compound that can be tested for its ability to kill cancer cells reminds us that the screening of natural products for discovering new drugs is in need of a revolution. Exploring the promising lead compounds from plants is quite a complex process, which requires a coordinated multidisciplinary research effort to develop new advanced analysis methods and technologies to extract, isolate, and identify potential compounds and then turn them into promising leads. With the sustained development of anticancer drug research and technological advances, such as high-throughput screening, molecular modeling-based drug design, the discovery of new anticancer drugs in plants will enter a new era in which natural products continue to play a major role.

For most bladder cancer patients, especially those who do not respond to specific anticancer drugs or have developed drug resistance, the development of novel therapeutic targets is a matter of urgency, and the discovery of traditional or non-specific anticancer drugs should continue. Any strategy for treating bladder cancer involves targeting affected cancer cells while minimizing the harmful effects on normal cells; with the ideal anticancer drug being only selectively cytotoxic to cancer cells. Phytochemicals found in plants and their derivatives are promising options for cancer therapy with improved treatment effects and reduced toxicity. Plant-derived compounds have clear advantages over conventional therapies because they are often multitargeted and possess relatively low toxicity, resulting in better treatment. Although extensive differences have been found in many studies, a number of agents of plant origin have shown significant potential against bladder cancers in different preclinical and clinical studies. This review summarizes the promising compounds for bladder cancer treatment and will hopefully rekindle the enthusiasm for discovering new therapies for bladder cancer based on natural products.
